# Plant-derived chitosan nanoparticles: antibacterial and improved crop performance under drought stress

**DOI:** 10.7717/peerj.21209

**Published:** 2026-05-28

**Authors:** Reham M. Aldahasi, Hessa O. Aldraiwiesh, Dhuha Fahad Alsuwaid, Kholoud A. Baeshen, Mudawi M. Nour, Modhi O. Alotaibi, Afrah E. Mohammed

**Affiliations:** 1 Microbiology and Immunology Unit, Natural and Health Sciences Research Center, Princess Nourah bint Abdulrahman University, Riyadh, Saudi Arabia; 2 Department of Biology, Princess Nourah bint Abdulrahman University, Riyadh, Saudi Arabia; 3 Habitat Regeneration and Landscaping, Wildlife and Natural Heritage, Royal Commission for AlUla, AlUla, Saudi Arabia; 4 Environmental and Biomaterial Unit, Natural and Health Sciences Research Center, Princess Nourah bint Abdulrahman University, Riyadh, Saudi Arabia

**Keywords:** PEG-induced osmotic stress, Drought simulation, Sustainable agriculture, Plant-derived bioactive compounds, Stress tolerance

## Abstract

This study presents plant-assisted synthesis of chitosan nanoparticles (CNPs) using *Adansonia digitata* extract via ionic gelation, demonstrating their dual role as antimicrobial agents and enhancers of seed germination under polyethylene glycol (PEG)-induced osmotic stress, a model for drought conditions. The CNPs were characterized by ultraviolet-visible spectroscopy, scanning electron microscopy (SEM), and transmission electron microscopy (TEM), and evaluated against *Escherichia coli, Staphylococcus aureus, and* methicillin-resistant *Staphylococcus aureus* (MRSA). Seed germination assays involved soaking seeds in a one mg/mL CNP solution for 12 hours. Results revealed that CNPs were efficiently produced via ionic gelation of chitosan with TPP in the presence of *A. digitata* extract, resulting in spherical particles with a peak absorption at 339 nm and an average size of 13.06 ± 0.05 nm. The nanoparticles exhibited strong antibacterial activity, particularly against *E. coli*, and significantly enhanced seed germination and growth under both normal and water-limited conditions. These findings highlight that integrating plant-derived bioactive compounds into chitosan nanoparticles not only boosts antimicrobial properties but also improves plant resilience to osmotic stress. This dual functionality positions plant-based CNPs as a sustainable strategy to address drought and pathogen-related challenges in agriculture.

## Introduction

Nanotechnology is becoming a significant area of research where the scientists are learning how to alter materials at the atomic and molecular scale to nano scale and comprehend their structure and behavior (Szczyglewska, Feliczak-Guzik & Nowak, 2023). Nanoparticles (NPs), materials at less than 100 nm in size, can be produced in a range of sizes and forms through physical, chemical, or biological fabrication (Rathod et al., 2024). The potential applications of nanotechnology in a wide range of fields, such as catalysis, gas sensing, renewable energy, electronics, medicine, diagnostics, medication delivery, cosmetics, the construction industry, and the food industry, have piqued the interest of scientists worldwide recently (Kumar, Kumar & Luthra, 2023). The key factors that determine the characteristics of NPs are their size and shape. The production of NPs with the right size, structure, monodispersity, and morphology is crucial for the aforementioned applications (Khan et al., 2022a; Khan et al., 2022b).

Further, chitosan is a natural polysaccharide derived from a wide variety of natural sources that has a wide range of uses in the culinary, cosmetics, pharmaceutical, and biomedical sectors (Pal et al., 2021). Chitosan can be derived from diverse sources, including crustacean shells (such as lobsters, crabs, and shrimp), fish scales, and various fungi and insects (Kumari & Kishor, 2020). Common extraction methods encompass enzymatic hydrolysis of crustacean biowaste, diverse fermentation processes, and conventional lactic fermentation bioprocesses (Pellis, Guebitz & Nyanhongo, 2022).

Beyond simply promoting growth, chitosan provides individual plants with multiple benefits, including antibacterial protection, enhanced immunity, and increased resistance to disease (Maluin & Hussein, 2020). It has been shown that the application of biocompatible materials, like chitosan, in seed technology enhances the germination process and seedling establishment (Godínez-Garrido et al., 2022). It has the ability to activate proteinase inhibitors, pathogenesis-related proteins, and phytoalexins for plants under stress. Chitosan, when applied to plants prior to exposure to abiotic stresses such as heat, salinity, and drought, enhances plant growth and stimulates the production of secondary metabolites and antioxidant enzymes (Malerba & Cerana, 2020). It is well known that using NPs is highly effective compared to their bulk materials due to the small size and high distribution area (Joudeh & Linke, 2022). Consequently, chitosan nanoparticles (CNPs) have garnered significant attention in recent decades as versatile and promising cationic polymeric NPs (Garavand et al., 2022). Their biocompatibility, biodegradability, environmental safety, and non-toxicity make CNPs ideal for a wide range of biological applications, particularly in the medical, agricultural, and pharmaceutical fields (El-Naggar et al., 2022b). In the fields of nanomedicine chitosan nanoparticles are extremely important (Sharifi-Rad et al., 2021). Chitosan faces challenges such as low adsorption efficiency and the use of toxic chemicals during synthesis. There’s a need for greener methods using natural crosslinkers and eco-friendly core approach incorporating biological components, which could enhance material performance (Muddin et al., 2024).

CNPs exhibit potent antimicrobial action against pathogenic multidrug-resistant bacteria, *A. baumannii, E. coli, S. aureus, P. aeruginosa,* and *K. pneumoniae* (El-Naggar et al., 2022a).

Due to their unique properties and the need for high production, CNPs could be prepared using eco-friendly approaches.

Plant extract-mediated nanoparticle synthesis and its potential applications in several sectors have been the subject of much investigation because of its low cost, nontoxic nature, easy availability, and eco-friendliness (Khan et al., 2022a; Khan et al., 2022b). Employing plant extracts in combination with sodium tripolyphosphate (TPP) is an emerging nanotechnology approach; they exert a synergistic effect rather than acting as reducing agents for production NP (Özdamar, Sürmeli & Şanlı-Mohamed, 2023). Plant metabolites, including sugars and antioxidants, play crucial roles in NP synthesis (El Shafey, 2020). These environmental friendly methods offer the potential to formulate nanoparticles with controlled size and shape for diverse applications (Naikoo et al., 2021). Various plant extracts, such as those from *Olea europaea* (olive) leaves (El-Naggar et al., 2023a). *Eucalyptus globulusLabill* fresh leaves (El-Naggar et al., 2022a). and *Lavandula angustifolia* (lavender) leaves (El-Naggar et al., 2023b) have been utilized in the biofabrication of CNPs. Furthermore, chitosan nanoparticles have been effectively employed over time in diverse applications to mitigate the negative effects of both biotic and abiotic stressors on plants. Seed priming, a key method among the nanoparticle application techniques, plays a crucial role in promoting plant development by triggering signaling pathways associated with the production of ROS and phytohormones (Etesami, Fatemi & Rizwan, 2021). The instances of chitosan and nano-chitosan are environmentally benign solid matrix priming agents that enhance seed germination under salt and other stressful conditions by greatly improving germination percentages and overall seedling health (Sen et al., 2020). Currently, *Petroselinum crispum* was tasted due to its many health advantages, which have been utilized in traditional medicine and a new study shows that its bioactive components may help to promote kidney health (Alobaidi, 2024).

Due to the characteristically low germination rate of *Petroselinum crispum* seeds in biological populations, significant research is focused on improving both their germination and overall growth (Mirjalili & Poorazizi, 2024). There is an application of Anatase nanoparticles (TiO_2_ NPs) on *Petroselinum crispum* seeds that increase germination rate (Dehkourdi & Mosavi, 2013)

The impact of CNPs on seed germination has been widely investigated. For instance, (Li et al., 2019) found that CNP-treated Wheat (*Triticum aestivum*) seeds exhibited higher germination. Similarly, Behboudi et al. (2019) demonstrated that CNPs enhanced germination in wheat under drought conditions. Therefore, the use of CNPs may promote growth and provide protection in early plant development

The CNPs enhance plant metabolic activity, as research indicates that they increase total phenolic content (Chandra et al., 2015), elevate photosynthetic rates (Ali et al., 2021), enhance chlorophyll and carotenoid levels, and improve mineral uptake (Arif, Siddiqui & Hayat, 2021). Additionally, CNPs mitigates the adverse effects of drought by boosting chlorophyll concentration, enhancing photosynthesis, increasing antioxidant enzyme activity, and improving relative water content (RWC), yield, and biomass (Attaran Dowom et al., 2022).

The mechanism chitosan and its nanoparticle form enhance seed germination involves a combination of physical, physiological, and biochemical processes. CNPs improve seed germination by facilitating water absorption during the imbibition phase and activating essential metabolic and antioxidant pathways. They increase the activity of nitrate reductase, reduce oxidative stress by lowering levels of hydrogen peroxide (H_2_O_2_) and malondialdehyde (MDA), and stimulate antioxidant enzymes. These effects contribute to a higher germination rate, increased seed vigor, and better early seedling growth (Behboudi et al., 2018; Allam et al., 2024).

In addition to their plant-growth-promoting effects, CSNPs exhibit potent antibacterial activity primarily through electrostatic interactions between their positively charged amino groups and negatively charged bacterial cell membranes. This disrupts membrane permeability, causing osmotic imbalances and leading to cell death. They can penetrate the bacterial cell wall, interfere with the electron transport chain, and bind to bacterial DNA, inhibiting replication, especially with low molecular weight chitosan. Additionally, they can flocculate electronegative elements within the cell, disrupting physiological functions and contributing to cell death (Chandrasekaran, Kim & Chun, 2020).

Chitosan nanoparticles (CNPs) are recognized to have antibacterial and plant growth promoting qualities, but little is known about how plant incorporating affects their surface chemistry and biological performance. As a sustainable bio-functionalizing agent during CNPs synthesis, *Adansonia digitata* extract is examined in this work. During ionic gelation, the extract is added as natural stabilizing and bio-functionalizing agent and not as a reducing agent. In order to determine if plant-derived metabolites can alter the surface chemistry and colloidal stability of CNPs while maintaining their known biological roles, this work is explored.

The African baobab (*Adansonia digitata* L.) is a multifunctional orphan tree species found in semi-arid and subhumid Sub-Saharan Africa, where it contributes significantly to rural lives. Africa’s food and nutrition security depends on this species because of its extensive range and high nutritional content (Assogbadjo et al., 2021). Baobab’s bioactive components, including phenols, flavonoids, proanthocyanins, tannins, catechins, and carotenoids, have been linked to its health advantages. Vitamin C and micronutrients including zinc, potassium, magnesium, iron, calcium, and protein are also abundant in baobab fruit, which may help prevent nutritional deficits (Silva et al., 2023). Various portions of the tree are used in African traditional medicine to cure a variety of diseases, including malaria, fever, diarrhea, microbiological infections, anemia, and toothache. Furthermore, different plant sections are utilized as food sources. Topically, the seed oil is used as a medication or cosmetic (Komane et al., 2023).

The objectives of the current study were to synthesize chitosan nanoparticles (CNPs) *via* an eco-friendly ionic gelation method incorporating bioactive compounds from *A. digitata* fruit shells. Such approach provided a plant-assisted surface functionalization step under mild aqueous conditions without toxic reagents. Further, their functionality as antimicrobial agents and drought stress mitigators were determined by (a) detecting CNPs efficacy against *E. coli*, *S. aureus*, and MRSA (b) evaluating their impact on seed germination under polyethylene glycol (PEG)-induced osmotic stress.

## Materials & Methods

### Materials

The fruit of *Adansonia digitata* purchased from the Sudanese market, chitosan powder purchased from Amazon Saudi Arabia. Chitosan is a renewable and biodegradable biopolymer that is generated from chitin. Sodium tripolyphosphate (TPP) bought from Desertcart, and sodium hydroxide (NaOH) and acetic acid were obtained from the Laboratory of Princess Nourah bint Abdulrahman University, Riyadh, Saudi Arabia

### The preparation of aqueous extracts

**Figure 1:**
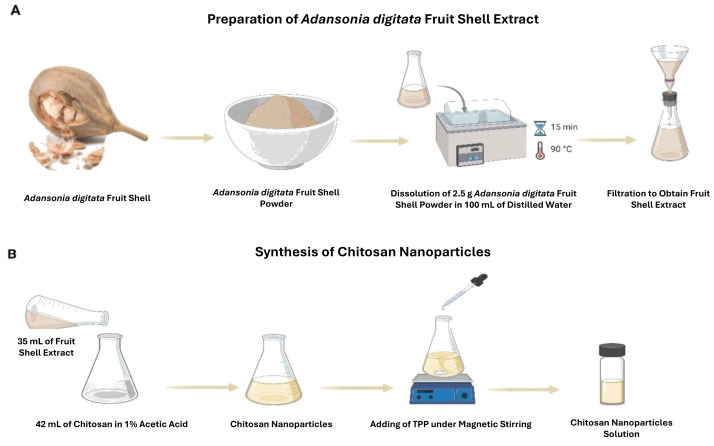
(A) The preparation of aqueous extract, and (B) synthesis of chitosan nanoparticles (CNPs). Created in BioRender (https://www.biorender.com/).

*Adansonia digitata* fruit shells were cleaned with distilled water to get rid of dust and other impurities, then dried at 70 °C for the entire night and ground into a fine powder for use in subsequent steps. To make an aqueous extract of the fruit shell of *A. digitata*, 2.5 g was dissolved to 100 mL of distilled water and heated to 90 °C for 15 min. Next, filter paper with a 20µm pore diameter was used to filter the extract as presented in Fig. 1A.

### Synthesis of chitosan nanoparticles

Chitosan nanoparticles (CNPs) were synthesized using the ionic gelation method with tripolyphosphate (TPP) as the crosslinking agent, as illustrated in Fig. 1B. Initially, chitosan powder was dissolved in 0.5% (v/v) acetic acid solution, adjusted to pH 5.1, to obtain a final concentration of three mg/mL. Then, 42 mL of this chitosan solution was added dropwise to 10 mL of TPP aqueous solution (0.007% w/v) under vigorous stirring at 1,000 rpm at room temperature. Subsequently, 35 mL of *A. digitata* shell extract was incorporated into the mixture and stirred thoroughly to ensure complete dissolution. Rather than providing enzymatic activity, *A. digitata* extract was used to give heat stable phytochemicals that serve as capping and stabilizing agents and may stay attached to the surface of the nanoparticle.

The entire experiment was conducted at room temperature. Finally, the resulting CNPs were collected by centrifugation at 14,800 rpm for 15 min. After collection, pellet was washed three times with deionized water at 4,500 rpm for 10 min to remove unbound phytochemicals.

### Characterization of CNPs

### Ultraviolet–visible spectroscopy

An analysis of UV-Vis spectra was carried out with an Evolution 201 UV-Visible spectrophotometer from Thermo Fisher Scientific, located in Waltham, MA, USA. The reaction mixture was examined over a wavelength range of 200 to 500 nm after 24 h, using distilled water as a comparison.

### Fourier-transform infrared spectroscopy

The functional groups in the phytoconstituents responsible that act as capping agents were analyzed using FTIR spectroscopy (SPECTRUM100; Perkin-Elmer, Waltham, MA, USA). The FTIR spectroscopy scanning covered a range from 500 to 4,000 cm.

### Scanning electron microscopy and transmission electron microscopy (TEM)

The scanning electron microscopy (SEM) instrument from JEOL in Tokyo, Japan, with added features of energy dispersive X-ray (EDX) and elemental mapping, was employed to analyze the surface characteristics, dimensions, and elemental makeup of metals present in biosynthesized nanoparticles. The transmission electron microscopy (TEM) equipment from JEOL in Tokyo, Japan, was utilized to analyze the size and shape of nanoparticles.

### *In-vitro* antibacterial assay

The disk diffusion technique described by Mohammed et al. (2024) , was used to investigate the antibacterial properties of the chitosan nanoparticles against clinical strains of *S. aureus*, *E. coli*, and MRSA. Freshly cultivated bacteria were employed in the synergistic antibacterial activity test; Nutrient agar was used as the growth medium(Ramzan et al., 2022). A volume of 0.2 mL of bacterial suspension (1.5 × 10^8^ CFU/mL) was uniformly swabbed onto individual agar plates using sterile cotton swabs. Antibiotic disks (six mm) were then supplemented with 100 µL of CNPs solution (150 mg/mL). Plates were incubated at 37 °C for 24 h, and the diameter of the inhibition zones (mm) surrounding the disks was measured to determine the combination’s synergistic antibacterial activity. Ampicillin was used as a positive control. All treatments were done in three replicates.

### Seed germination

The procedures were followed, and some concentrations were adjusted appropriately (Allam et al., 2024). The *Petroselinum crispum* seeds were sterilized for 15 min in a diluted solution of 2% sodium hypoclorite before being carefully cleaned four times with distilled water (El-Kenany et al., 2017) then let to air dry. Seeds were immersed for twelve hours in CNPs solution (one mg/mL), and an equal number of seeds were soaked in water as a control. After being soaked, the seeds were washed with distilled water and allowed to air dry between two filter papers.

### Germination bioassay

Petri plates were used to allow primed seeds to germinate, eight seeds were kept on filter paper in each dish and watered every day with four mL of distilled water. To compare control and treated seeds and assess the biological effect of chitosan nanoparticles on the percentage of *Petroselinum crispum seed* germination. Furthermore, in a trial to understand the effect of these socking process in enhancing germination under drought stress, seeds were watered by four mL of polyethylene glycol (PEG) at a potential −1.2% all trials were done in three replicates. Every day the seeds were monitored, and the number of germinated seeds was recorded. After 6 days the length of seeds was measured using the ruler and the germination rate was calculated using the following formula:

\begin{eqnarray*}\mathbf{Germinationrate}= \frac{\text{Total number of germinated seeds}}{\text{Total number of seeds}} \times 100. \end{eqnarray*}

### Statistical analysis

Statistical analysis was performed using one-way analysis of variance (ANOVA). Data are presented as mean ± standard deviation from three independent replicates, and error bars are shown. GraphPad Prism version 10.0.2.232 (GraphPad Software, La Jolla, CA, USA) was used for statistical analyses. FTIR and UV-Vis spectra were generated using OriginPro^®^ 2023b, and the size and diameter of the particles were measured from TEM images using ImageJ software.

## Results

### Ultraviolet-visible spectroscopy

**Figure 2:**
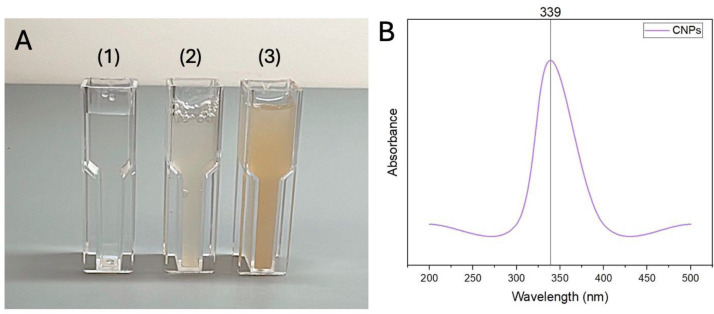
(A) 1 - chitosan solution; 2 - chitosan with *A. digitata*; 3 - chitosan nanoparticles (CNPs), (B) UV analysis of CNPs prepared using *A. digitata* fruit shell extract.

UV absorbance was measured when the color changes in the reaction media (*A. digitata* fruit shell extract + chitosan with TPP) were noted, indicating NPs formulation. Figure 2 shows the absorbance peaks of CNPs at 339 nm.

### Fourier transforms infrared measurements

**Figure 3:**
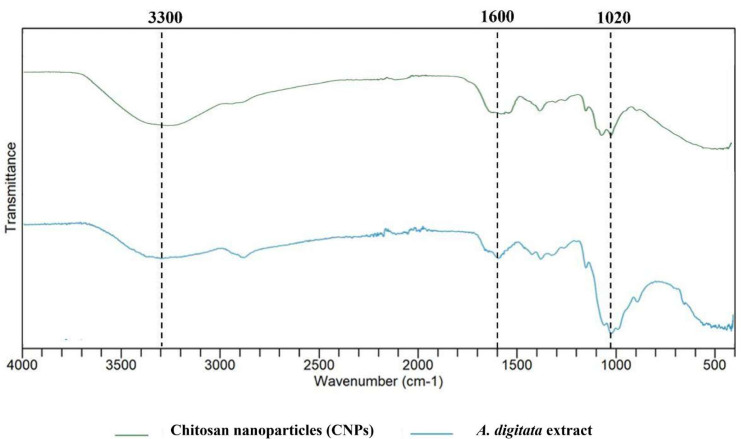
FTIR analysis of chitosan nanoparticles (CNPs) and *A. digitata* fruit shell extract.

Fourier transforms infrared (FTIR) analysis was carried out to determine the probable bioactive phytochemicals in *A. digitata* fruit shell extract. FTIR spectra of the synthesized CNPs is displayed in Fig. 3. The FTIR analysis displays the numbers of predominant peaks at 3,300, 1,600, and 1,020 cm^−1^ associated with numerous functional groups such as OH, amine group and glycosidic linkage stretching respectively. Given that peak locations and intensity changes indicate nanoparticle production and plant metabolites can remain adsorbed on the nanoparticle surface as capping agents, similarities between the extract and CNP spectra are expected.

### Scanning electron microscopy and transmission electron microscopy

**Figure 4:**
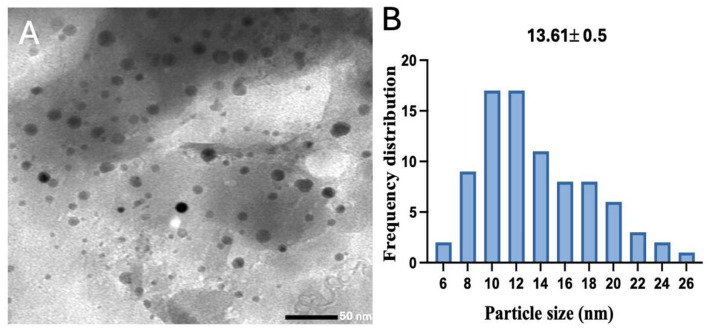
(A) TEM image of the synthesized CNPs, and (B) their frequency distribution with mean particle size. Size measurements were analyzed by ImageJ software constructed from TEM micrographs at scale bars: 100 nm.

TEM analysis indicated a spherical morphology of CNPs, as demonstrated in Fig. 4A, and the size diameter of CNPs was 13.61 0.5 nm, as shown in Fig. 4B.

**Figure 5:**
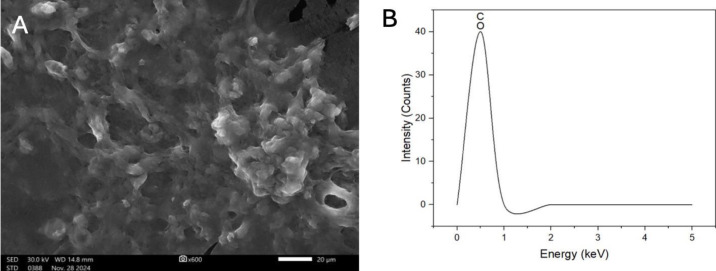
(A) SEM image of fabricated CNPs, and (B) their EDX analysis indicating the elemental composition.

On the other hand, the SEM image indicated a smooth surface and a uniform shape in Fig. 5A. Furthermore, the elemental makeup by EDX reported peaks at 0.5 KeV for C and O in Fig. 5B.

### Antibacterial activity

**Figure 6:**
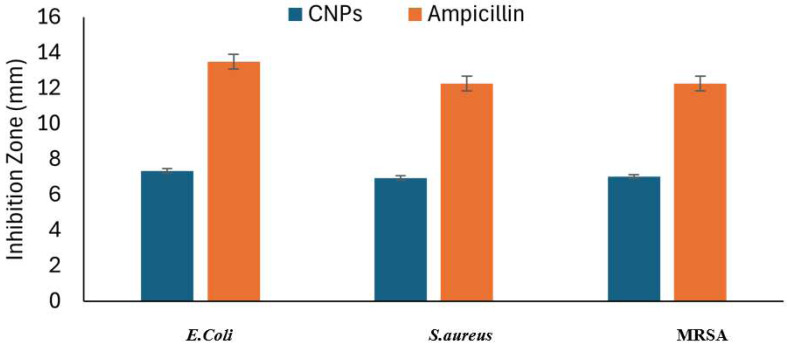
Activity of chitosan nanoparticles (CNPs) compared to ampicillin.

CNPs had potent antibacterial activity against the tested bacterial *E. coli* with clear inhibition zones 7.34 ± 0.47 mm, *S. aureus* 6.93 ± 0.29 mm, and MRSA 7.01 ± 0.43 mm. Ampicillin indicated inhibition zone of 13.5 ± 0.5, 12.25 ± 0,5 and 12.25 ± 0,5 respectively as shown in the Fig. 6.

### Seed germination

**Figure 7:**
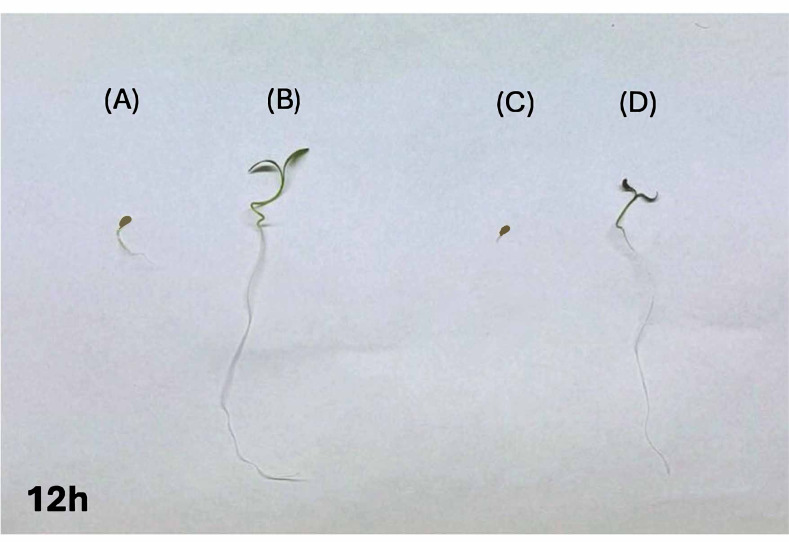
Growth performance of *Petroselinum crispum* seeds under different treatments: (A) seeds soaked in water, (B) seeds soaked in CNPs, (C) seeds soaked in water and grown under PEG-induced stress, and (D) seeds soaked in CNPs and grown under PEG-induceds.

Soaking *Petroselinum crispum* seeds for 12 h in a CNPs solution (one mg/mL) significantly enhanced germination compared to the control and alleviated the inhibitory effects of PEG-induced stress (Fig. 7). CNPs-treated seeds exhibited higher germination percentages (GP%) than untreated controls under both water and PEG conditions.

**Figure 8:**
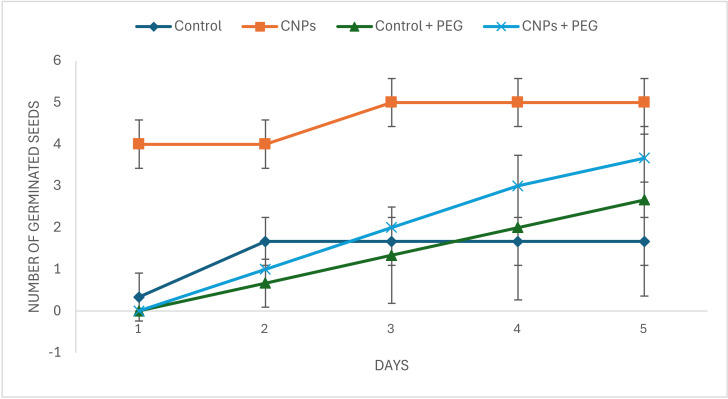
Effect of chitosan nanoparticles (CNPs) on germination of *Petroselinum crispum* seeds under normal and PEG-induced osmotic stress over five days. Data are presented as mean ± SD (*n* = 3).

Over a five-day period (Fig. 8), control seeds germinated gradually, reaching germination by day four, whereas CNPs-treated seeds achieved maximum germination by day three. PEG stress delayed and reduced germination in untreated seeds, but CNP pretreatment partially mitigated this effect, resulting in improved germination under stress, though slightly below control levels under normal conditions.

**Figure 9:**
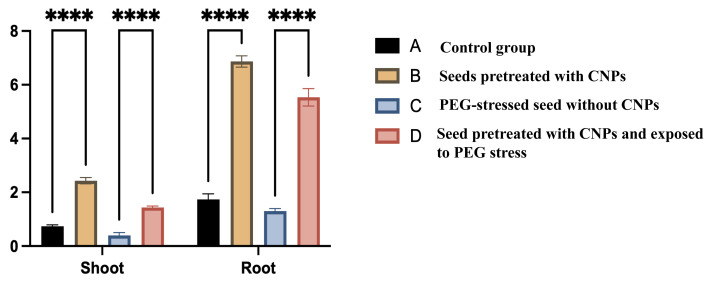
The length (cm) of shoots and roots of seeds pre-treated by CNPs for 12 h, where is control, for the seeds pretreated by CNPs is for the seeds under PEG stress and is for the seeds pretreated with CNPs under PEG stress.

These findings suggest that CNPs promote seed germination and enhance seedling viability by reducing the adverse effects of osmotic stress. Additionally, CNP pretreatment positively influenced shoot and root growth under both normal and PEG-stressed conditions (Fig. 9).

### Shoot length

In comparison to the control group (Group A), the seeds prepared with CNPs (Group B) exhibited a notable increase in shoot length. Comparing Group C to the control, PEG stress resulted in a sharp decrease in shoot length. In contrast to PEG-stressed seeds without CNP pretreatment (Group C), seeds pretreated with CNPs and exposed to PEG stress (Group D) showed a significant increase in shoot length, indicating a protective effect of CNPs against stress.

### Root length

Similarly, seeds pretreatment with CNPs (Group B) showed a significant increase in root length when compared to the control group (Group A). Root length was significantly reduced as a result of PEG stress (Group C). When compared to seeds exposed to PEG stress without CNP pretreatment (Group C), pretreatment with CNPs under PEG stress conditions (Group D) markedly increased root length.

Overall, our findings suggest that CNPs improve seed tolerance to stress by promoting shoot and root development in normal circumstances and reducing the adverse effects of PEG-induced stress.

## Discussion

In this study, CNPs were fabricated *via* ionic gelation using *A. digitata* fruit shell extract. Plant- may add bioactive phytochemicals to the surface of the CNPs that potentially affecting their stability and biological interactions (Ying et al., 2022; Thatyana et al., 2023). The conventional synthesis of nanoparticles often relies on physical and chemical methods, which pose significant environmental and biological hazards due to their toxicity and harmful reagents. In contrast, biological synthesis offers a sustainable, cost-effective, and eco-friendly alternative, making it a highly promising approach (Kuppusamy et al., 2016).

Plants have been extensively utilized as an eco-friendly method of nanoparticles formation due to their rich phytochemical composition, which acts as natural reducing and stabilizing agents (Ying et al., 2022; Thatyana et al., 2023). Several studies have successfully employed plant extracts in nanoparticle synthesis, including those derived from *Citrus limon* (lemon), *Ginkgo biloba*, *Pelargonium graveolens*, and tea extracts (El-Naggar et al., 2022a; Owczarek et al., 2023; Sathiyabama et al., 2024; Rahman et al., 2024).

The current study absorption peak was detected at 339 nm. This result aligns with (Sathiyabama et al. (2024). Also, CNPs biosynthesized using aqueous extract from *Lavandula angustifolia* leaves showed a peak at 285 nm (El-Naggar et al., 2023b). On the other hand, Biosynthesized CNPs using green tomato extract showed an absorbance peak at 363 nm(Abdallah et al., 2020).

FTIR analysis highlights the structural stability of chitosan during its conversion into CNPs.

The FTIR spectra of extract and CNPs’ are comparable, indicating that the phytochemicals from *A. digitata* stayed attached to the nanoparticle surface and functioned as natural stabilizing and capping agents instead of enzyme reducers. This is confirmed by the retention of key peaks such as –OH in both the extract and CNPs, indicating that no significant chemical changes occurred during synthesis (Pasieczna-Patkowska, Cichy & Flieger, 2025). In this study, the FTIR spectra of CNPs exhibited absorption peaks at 3,300, 1,600, and 1,020 cm^−1^. The broad band around 3,300 cm^−1^ corresponded to O-H stretching vibrations, indicating the presence of hydroxyl groups from alcohols (Abdallah et al., 2020). The peak at 1,600 cm^−1^ was attributed to amine group vibrations (El-Naggar et al., 2023b), while the absorption band at 1,020 cm^−1^ was associated with the glycosidic linkage stretching (Sathiyabama et al., 2024), confirming the structural characteristics of chitosan nanoparticles. Additionally, the presence of intense bands in the FTIR spectra confirms the role of a capping agent in stabilizing the nanoparticles and preventing aggregation during colloidal synthesis (Abdallah et al., 2020). Plant metabolites like polyphenols and flavonoids, are thought to be responsible only for the stabilization of nanoparticles (Kesharwani et al., 2009) because thermal treatment during synthesis reduces enzymatic activity. Further, such metabolites from the extract may interact with chitosan *via* electrostatic interactions during nanoparticle formation (Gaetano et al., 2025).

The optimization conditions for the fabrication process of CNPs utilizing *A. digitata* fruit shell extract were followed, and the resulting surface morphology of the obtained CNPs was analyzed. The SEM image revealed a favorable dispersion and spherical shape of the nanoparticles. In comparison, the phyto-fabricated CNPs derived from green tomato extract also exhibited a spherical morphology (Abdallah et al., 2020). Furthermore, the TEM image indicated that the CNPs displayed a high degree of agglomeration, presenting an irregular spherical shape. In a related study, bio-synthesized CNPs produced using *Lavandula angustifolia* leaf extract demonstrated irregular shapes, with a size of 9.77 nm (El-Naggar et al., 2023b).

The CNPs exhibited antibacterial activity against the tested bacterial pathogens in the following order: *E. coli*, *S. aureus*, and MRSA. These findings align with the study by Duraisamy et al. (2022), which demonstrated that *Martynia annua* extract, combined with chitosan nanoparticles, showed significant antibacterial effects against *E. coli* and *S. aureus*, particularly at higher concentrations ranging from 50 to 500 µg/mL. Similarly, another study reported by Rahman et al. (2024) that CNPs synthesized with lemon extract displayed notable antibacterial activity against *E. coli*, *S. aureus*, and MRSA at a concentration of one mg/mL. The enhanced antimicrobial efficacy of CNPs can be attributed to their phytochemical composition and the inherent properties of chitosan which might disrupts bacterial cell membranes, ultimately causing bacterial cell death (Alamgir, 2018; Yan et al., 2021).

Further, the effects of CNPs at a concentration of one mg/mL on the germination of *Petroselinum crispum* seeds were assessed for the first time to germination percentage and seedling length. The results demonstrated a significant improvement in both parameters compared to untreated controls in the normal condition and under PEG stress. These findings align with a previous study on wheat (*Triticum aestivum* L.), which demonstrated that CNPs treatment enhanced seed germination, likely due to the nanoparticles’ ability to modulate physiological and biochemical pathways involved in stress responses (Li et al., 2019). Similarly, a study indicates the beneficial effects of CNPs on seed germination and early plant development. For instance, a study on *Vicia faba* seeds primed with 0.05% and 0.1% CNPs solutions for 6 h reported improved germination rates and seedling growth, attributed to increased water uptake and activation of metabolic processes essential for germination (Abdel-Aziz, 2019). On the other hand, a prior study conducted by Bakhoum, Sadak & Tawfik (2022) revealed that drought stress notably elevated total soluble sugars, proline, and free amino acids, along with phenolic and flavonoid levels in lupine seeds. In another study by Ajmal et al. (2023), authors reported notable germination and growth of *Vigna radiata*, highlighting its adaptability responses to NPs treatment under PEG-induced drought stress. The positive effects of CNPs nano-priming may be due to enhanced water absorption, activation of germination metabolism, and promotion of better seedling growth (Li et al., 2019; Allam et al., 2024). Additionally, CNPs may facilitate nutrient uptake and reinforce cell wall integrity, thereby improving seedling establishment under unfavorable conditions (Van Nguyen, Dinh Minh & Nguyen Anh, 2013).

Overall, this study reinforces the growing evidence that CNPs are an effective seed priming agent, enhancing germination and early seedling development across diverse plant species. It supports their application as an eco-friendly and effective approach to improving seed germination and stress resilience in crops. In addition, the antibacterial activity of CNPs against *E. coli*, *S. aureus,* and MRSA indicates that using them as seed priming agents could also help reduce the risk of foodborne pathogens. This dual benefit shows that CNPs can both support seed germination and seedling growth and enhance postharvest food safety. Future research will include a direct side-by-side comparison with traditionally synthesized CNPs made without plant extract (normal ionic gelation) in order to measure the precise role that *A. digitata* plays in the surface characteristics and bioactivity of nanoparticles.

## Challenges and Limitations

Despite promising results, several challenges remain. Synthesizing chitosan nanoparticles *via* ionic gelation in the presence of *Adansonia digitata* extract requires careful control to ensure consistent size and shape, and natural variation in the extract can affect bioactivity. Antimicrobial testing and seed germination assays also demand precise conditions to produce reliable results. Translating these findings to real-world agricultural settings adds further complexity, as soil variability, microbial interactions, and environmental fluctuations may influence nanoparticle performance. Addressing these challenges will be key to realizing the full potential of plant-assisted CNPs for sustainable agriculture.

## Conclusions

This study demonstrates a plant-assisted, eco-friendly synthesis of chitosan nanoparticles *via* ionic gelation using *Adansonia digitata* fruit shell extract, offering a sustainable approach. Characterization confirmed their structural integrity and favorable morphology, and antimicrobial assays showed significant activity against *E. coli, S. aureus, and* MRSA. CNPs also enhanced germination of *Petroselinum crispum* seeds under stress, indicating their role in supporting key physiological processes. By combining antimicrobial protection with growth promotion, plant-derived CNPs offer a dual-function approach with strong potential for sustainable agriculture.

Future work should address challenges including variability in plant extract composition, optimization of synthesis parameters for reproducibility, and validation under field conditions, where environmental factors and soil interactions may affect nanoparticle performance. Overcoming these hurdles will be essential for translating laboratory findings into practical agricultural applications.

## Supplemental Information

10.7717/peerj.21209/supp-1 Supplemental Information 1 Antibacterial activity

10.7717/peerj.21209/supp-2 Supplemental Information 2 Seed germination

## Additional Information and Declarations

### Data Availability

The following information was supplied regarding data availability: The raw data are available in the Supplemental Files.
